# Gut Microbiome Dysbiosis and Immunometabolism: New Frontiers for Treatment of Metabolic Diseases

**DOI:** 10.1155/2018/2037838

**Published:** 2018-12-09

**Authors:** José E. Belizário, Joel Faintuch, Miguel Garay-Malpartida

**Affiliations:** ^1^Department of Pharmacology, Institute of Biomedical Sciences, University of São Paulo, SP CEP 05508-900, Brazil; ^2^Department of Gastroenterology of Medical School, University of São Paulo, SP CEP 05403-000, Brazil; ^3^School of Arts, Sciences and Humanities (EACH), University of São Paulo, São Paulo, SP CEP 03828-000, Brazil

## Abstract

Maintenance of healthy human metabolism depends on a symbiotic consortium among bacteria, archaea, viruses, fungi, and host eukaryotic cells throughout the human gastrointestinal tract. Microbial communities provide the enzymatic machinery and the metabolic pathways that contribute to food digestion, xenobiotic metabolism, and production of a variety of bioactive molecules. These include vitamins, amino acids, short-chain fatty acids (SCFAs), and metabolites, which are essential for the interconnected pathways of glycolysis, the tricarboxylic acid/Krebs cycle, oxidative phosphorylation (OXPHOS), and amino acid and fatty acid metabolism. Recent studies have been elucidating how nutrients that fuel the metabolic processes impact on the ways immune cells, in particular, macrophages, respond to different stimuli under physiological and pathological conditions and become activated and acquire a specialized function. The two major inflammatory phenotypes of macrophages are controlled through differential consumption of glucose, glutamine, and oxygen. M1 phenotype is triggered by polarization signal from bacterial lipopolysaccharide (LPS) and Th1 proinflammatory cytokines such as interferon-*γ*, TNF-*α*, and IL-1*β*, or both, whereas M2 phenotype is triggered by Th2 cytokines such as interleukin-4 and interleukin-13 as well as anti-inflammatory cytokines, IL-10 and TGF*β*, or glucocorticoids. Glucose utilization and production of chemical mediators including ATP, reactive oxygen species (ROS), nitric oxide (NO), and NADPH support effector activities of M1 macrophages. Dysbiosis is an imbalance of commensal and pathogenic bacteria and the production of microbial antigens and metabolites. It is now known that the gut microbiota-derived products induce low-grade inflammatory activation of tissue-resident macrophages and contribute to metabolic and degenerative diseases, including diabetes, obesity, metabolic syndrome, and cancer. Here, we update the potential interplay of host gut microbiome dysbiosis and metabolic diseases. We also summarize on advances on fecal therapy, probiotics, prebiotics, symbiotics, and nutrients and small molecule inhibitors of metabolic pathway enzymes as prophylactic and therapeutic agents for metabolic diseases.

## 1. Introduction

Human microbiomes refer to collective genomes of bacteria, archaea, viruses, protozoans, and fungi that coinhabit multiple ecosystems in the human body (Bäckhed et al. [1], Belizario and Napolitano [2]). An adult man of 70 kg might contain up to 3.8 × 10^13^ bacteria, which is equal to the number of cells of an adult human body (Sender et al. [3]). Bacteria are morphologically and biochemically classified based on various properties including wall type, shape, requirement of oxygen (anaerobic or aerobic), endospore production, motility, and their metabolism. Bacteria are also classified by phylogenetic diversity of variable nucleotide sequences of small subunit ribosomal RNA operons or 16S and 18S rRNA genes [1, 2].

The Human Microbiome Project (HMP) have been defining criteria for high-quality and comprehensive metagenomic analysis of genetic material recovered directly from distinct sites on the human body to determine the microbial relative abundance of multiple strains and species of different phyla at physiological conditions [4–6]. Advances on computational techniques have allowed studies of public human metagenomes based on the phylogenetic clustering and assembly of bacterial genomes into taxonomic domain, kingdom, phylum, class, order, family, genus, and species [4–8]. The analyses of various human microbiome datasets revealed the immense diversity at both populations and individuals over evolutionary and lifetime [7–9]. The gut microbiota of healthy individuals is composed of permanent and transitory microbial species and subspecies of over 17 candidate bacterial phyla belonging to Firmicutes (>70%), Bacteroidetes (>30%), Proteobacteria (<5%), Actinobacteria (<2%), Fusobacteria and Verrucomicrobia (<1%), and other phyla. The novel bacterial genome assembly and taxonomic profiling based on 1550 metagenome-assembled genomes (MAGs) have revealed nearly 70,000 bacterial and archaeal genomes and new species that are under deep investigation [6–8]. A common set of prevalent microbial species found in normal human stools includes the *Clostridiales* species such as *Coprococcus*, *Ruminococcus*, *Eubacterium*, *Bacteroides dorei* and *fragilis*, and *Alistipes finegoldii* and *onderdonkii* [7, 8]. The fully broad taxonomic distribution, microbial evolution, and metabolism of bacterial species regarding caloric load and nutrient absorption have served to cluster species-level phylotypes into major human enterotypes [9–12]. The prevalent human enterotype type 1 is characterized by high levels of *Bacteroides* and type 2 by few *Bacteroides* but high levels of *Prevotella*. They are, respectively, associated with individuals that ingest either high content of animal protein (type 1) or carbohydrates (type 2).

The gastrointestinal (GI) tract possesses its own nervous system known as the enteric nervous system. This system communicates with the central nervous system through nerves, such as the vagus, neuromodulators, and neurotransmitters of sympathetic and parasympathetic branches of the autonomic nervous system [1]. Bacterial richness and diversity in the GI microbiota occupy a central role in normal metabolic and immunological functions of tissues and organs [1, 2]. Here, we will update on diverse studies exploring the roles of microbiomes, nutrients, and metabolites on immune cell function, etiology of inflammatory, and metabolic diseases, including their wide range of mechanisms. We also describe why and how novel dietary and pharmacological strategies including fecal transplantation, probiotics, prebiotics, and small molecules may help to treat and modulate species-level phyla types and promote the restoration of microbiomes causing metabolic syndromes.

## 2. Gut Microbiota Controls the Host's Metabolic Physiological States

Microorganisms in the gut perform their functions largely through enzyme pathways, in order to digest complex dietary carbohydrates and proteins [13, 14]. Gut microbiota provides the branched-chain amino acids leucine, isoleucine, and valine, and particularly glycine, which is required for the synthesis of glutathione—the main intracellular antioxidant and detoxifying agent necessary for many biological functions of the host. Bacteria of the gut synthesize a large variety of signaling molecules of low molecular weight that include methane, hydrogen sulfide, and nongaseous metabolites [13, 14]. Those products are able turn on or off both host genes and microbe virulence and metabolism genes. Microorganisms also sense diverse environmental signals, including host hormones and nutrients, and respond to them by differential gene regulation and niche adaptation [13, 14].

The maintenance of a stable, fermentative gut microbiota requires diets rich in whole plant foods, particularly rich in fibers [13, 14]. These substrates are processed by the intestinal microbiota enzymes, such as glycoside hydrolases and polysaccharide lyases to produce polyamines, polyphenols, and vitamins B and K. Under anaerobic conditions, species belonging to the *Bacteroides* genus, and to the *Clostridiaceae* and *Lactobacillaceae* families, in special, *Citrobacter* and *Serratia* strains, produce short-chain fatty acids (SCFAs) which are volatile fat acids able to cross the blood-brain barrier via monocarboxylate transporters. SCFAs produced by intestinal bacteria are acetate (2 carbon atoms), propionate (3 carbon atoms), and butyrate (4 carbon atoms), and their molar ratios vary from 3 : 1 : 1 to 10 : 2 : 1, respectively. Most of SCFAs are metabolized to CO_2_. Butyrate acts on colonocytes, goblet cells, and Paneth cells and provides energy for cellular metabolism and regulates apoptosis, cellular differentiation, and chemical modification of nuclear proteins and nucleic acid. Acetate and propionate pass into the bloodstream and are taken up by the liver and peripheral organs, where they can act as substrates for gluconeogenesis and lipogenesis [14, 15]. The G-protein-coupled receptors (GPRs), GPR41 and GPR43, also named free fatty acid receptors 2 and 3 (FFARs 2/3) present in many tissues, including adipose, gut enteroendocrine cells, and inflammatory cells act as major receptors of SCFAs [14, 15]. Under certain physiological conditions, SCFAs can induce the secretion of glucagon-like peptides (GLP-1 and GLP-2) and peptide YY (PYY). GLP1 stimulates *β* cells of the pancreas to produce insulin, whereas PYY inhibits nutrient absorption in the intestinal lumen as well as control the appetite. The gut microbiota contributes to fat deposition through the regulation of the nuclear farnesoid X receptor (FXR), the bile acid receptor that is responsible for the regulation of bile acid synthesis, and hepatic triglyceride accumulation [16]. Bile acids, for example, deoxycholic acid, have antimicrobial effects on microbes of the gut and also induce the synthesis of antimicrobial peptides by gut epithelial tissue [17]. Moreover, microbiota converts carnitin and choline to trimethylamine and thus regulates directly the bioavailability of choline and indirectly the accumulation of triglycerides in the liver. The gut microbiota also helps the absorption of calcium, magnesium, and iron. High or low productions of SCFAs, tryptophan metabolites, GABA, noradrenaline, dopamine, acetylcholine, and 5-hydroxytryptamine (serotonin) are associated with various inflammatory and metabolic diseases and neuropsychiatric disorders [18]. Some of these factors act as major neurotransmitters and modulators of the brain-gut axis, and serotonin has central roles in sexuality, substance addiction, appetite, emotions, and stress response [18].

Thousands of microbiota-derived metabolites with known and unknown functions have been identified as components of the human metabolome [19–21]. GI microbiota produces large quantities of epigenetically active metabolites, such as folate and A and B vitamins (including riboflavin (B_2_), niacin (B_3_), pantothenic acid (B_5_), pyridoxine (B_6_), folate (B_9_), and cobalamin (B_12_)) that regulate the activity of host chromatin-modulating enzymes and genetic responses to environmental signals [22]. Acetyl-CoA produced by a number of metabolic processes is the acetyl donor for histone modification (acetylation and deacetylation) catalyzed by histone acetyltransferases. Glycine, serine, and methionine are substrates for DNA methylation and demethylation enzymes. Therefore, changes in gut microbiota can result in epigenomic changes not only directly in adjacent intestinal cells but also in distant cell lineages, such as hepatocytes and adipocytes [22]. Finally, bacteria can inhibit the growth of their competitors by long distance microbial communication, via release of metabolites and quorum sensing peptides, which are considered a biological strategy for maintenance of density of commensal species, and elimination of pathogenic bacteria [23, 24].

## 3. Gut Microbiota Drives Host Immunological Functions

A collection of 100-400 × 10^12^ of bacteria inhabits and lives in mutualistic relationships in the human GI tract [3]. The GI double mucus layer is formed by heavily O-glycosylated mucin proteins encoded by MUC2 gene of the mucin protein family. Most of the bacteria of the colon are tightly attached to the outer mucus layer, and the inner layer forms a physical barrier that limits bacterial contact with the epithelium. Most of microbial species in GI tract are transmitted at early life to babies through mother's milk, which contains predominantly *Bifidobacteria* and *Lactobacillus* species [25, 26]. Along the transition of infancy to adult life, increase in food sources drives the complexity and diversity of bacterial communities of the genera *Bacteroides*, *Parabacteroides* (Bacteroidetes), and *Clostridium* (Firmicutes) [1, 2, 25, 26]. Bacterial density in the jejunum/ileum (<10^5^) and in the large intestine progressively increases in comparison with the stomach and duodenum, and the highest taxa and cell density are present in the colon, which contains 10^9^-10^12^ colony-forming units per ml (99% of total GI population). They are anaerobes such *Bacteroides*, *Porphyromonas*, *Bifidobacterium*, *Lactobacillus*, and *Clostridium*. Anaerobic bacteria and oxygen-sensitive microbes are capable of producing of short-chain fatty acids than facultative aerobic bacteria such as *E. coli*, by a factor of 1000. Disruption of the dynamic interrelation between the host and the microbial communities causes dysbiosis, which is a bacterial imbalance between aerobic and facultative anaerobic bacteria ratios [1, 2]. Hypoxia prevents the growth of pathogenic facultative anaerobes such as *E. coli* and *Salmonella*. The rupture of gut barrier provoked by dysbiosis leads to local and systemic inflammation [1, 2, 27]. A study by Byndloss et al. unequivocally demonstrated that dysbiosis could be caused by high levels of oxygen and nitrates, which are compounds that contribute to the growth of *Escherichia* and *Salmonella species* [28]. Many diseases, including inflammatory bowel diseases (IBDs), irritable bowel syndrome (IBS), diabetes, obesity, and cancer, have been associated with specific bacterial dysbiosis [29–31]. The potential temporary shifts of microbiota species, for example, by the reduction of anti-inflammatory species, such as *Faecalibacterium prausnitzii* and *Akkermansia muciniphila* which predominate in healthy individuals, or by the proliferation of potentially proinflammatory bacteria such as *Bacteroides* and *Ruminococcus gnavus*, can promote the disease progression and chronicity [29–32]. Comparative studies in lean and obese animal models have indicated that low Bacteroidetes/Firmicutes ratio is a hallmark of obesity [33]. Firmicutes rely heavily on dietary carbohydrates, whereas Proteobacteria rely on proteins as carbon source. For instance, *Akkermansia muciniphila* is a mucin-degrading bacterium in the phylum of *Verrucomicrobia* that is present in great abundance in healthy humans, but is present in reduced number in patients with inflammatory and gastrointestinal diseases, obesity, and type 2 diabetes (T2D) [34]. In fact, distinct microbial communities and mechanisms can drive host's susceptibility to diseases and influence on clinical outcomes [35].

The gut microbiota plays a critical role in the immune system by controlling the development and functionality of gut-associated lymphoid tissues (GALT), including Peyer's patches, isolated lymphoid follicles, and mesenteric lymph nodes [30, 31, 36, 37]. Microbes and their products are necessary for the immune system to distinguish self from nonself (invaders) at early life and activation and maintenance of innate hematolymphoid cells (ILC1, 2, and 3), natural killer (NK) cells, and cytotoxic and noncytotoxic and helper lymphoid cells [36–39]. NK cells and ILC1 produce large amounts of IFN-*γ*, antimicrobial peptides (AMPs), granulysin, defensins, lysozyme, and Reg III*γ*, which together play critical functions on the regulation of microbial ecology and immune surveillance [24, 31, 39, 40]. For instance, polysaccharide A, *α*-galactosylceramide, and tryptophan metabolites produce by microbial communities stimulate immune cells to produce interleukin-22, Reg3*γ*, IgA, and interleukin-17 [37]. IgA is one important component of innate response to prevent invasion of the microorganisms into circulation [30]. T helper (Th) 17 cells and regulatory T cells (Treg) are antigen-specific populations that respond to transforming growth factor-*β* and retinoic acid and control immune tolerance [38, 39]. This control of whole body immune system by gut bacteria appears to be a delicate framework since loss of a specific species can lead to overreaction or suppression of the innate immune response [27, 35, 36].

A variety of membrane and intracellular receptors named “pattern recognition receptors” or PRRs expressed on the epithelial and immune cells act as sensors of bacterial and cellular products, which are named the pathogen-associated molecular patterns (PAMPs) and damaged-associated molecular patterns (DAMPs) [41, 42]. PAMPs and DAMPs such as lipopolysaccharides (LPS), lipid A, peptidoglycans, flagellin, microbial RNA/DNA, as well as host cell constituents, such as uric acid, HMGB1 (high-mobility group box 1 protein), double-stranded DNA, and mitochondrial, are recognized by the members of the Toll-like receptor (TLR) family and nuclear oligomerization domain-like receptor of NOD/NLR family [41, 42]. Extracellular and intracellular complexes formed by DAMPs and PAMPs and NOD/NLR receptors constitute the inflammasomes [43]. These cytosolic complexes associate with the adapter protein ASC (apoptosis-associated speck-like protein) and proinflammatory proteases of caspase family caspase 1, caspase 11, caspase 4, and caspase 5 [43]. Following the inflammasome activation occurs the production of interleukins IL-1*β* and IL-18. These cytokines increase the synthesis of other cytokines such as TNF-*α*, IL-6, IL-17, IL-22, and IL-23 and several active chemical inflammatory mediators [43].

Various studies using gene-deficient mice models have defined the direct and complex interplay of bacterial dysbiosis and genetic and environmental factors [35, 36, 44]. Many inflammatory diseases are caused by mutations or loss of some innate response genes in lymphoid tissues and smaller Peyer's patches and mesenteric lymph nodes [38, 39]. The components of inflammasomes such as MyD88, TLRs, NODs, NLrp3/6, ASC, and caspase 1 and caspase 11 are known to play a control of the intestinal dysbiosis [29, 38, 39, 45, 46]. For instance, NOD1^−^/^−^ and NOD2^−^/^−^ transgenic mice have increased susceptibility not only to inflammatory bowel disease but also to type 1 diabetes and cancer [38, 39, 45, 46]. The animal housing conditions and diet-induced microbiota composition are some examples that may be responsible for strain phenotypic differences in transgenic animals [47, 48]. Germ-free (GF) mice display underdeveloped lymphoid tissues, impairment of T and B cell function, and decreased CD4^+^ T cells and antibody production. Their Th17 and Treg cells are less efficient in the control of infection. Colonization of GF mice with limited number of bacterial species (gnotobiotic mouse models) can restore immunological functions [47, 48]. The phenotypes observed in these mice models are not always observed in human studies. It is important to mention that 85% of the murine microbiome species have not been detected in human microbiomes [49, 50]. Furthermore, the humanization of mouse models with human cells or human microbiota cannot adequately display the whole spectrum of relevant human disease phenotypes [47, 48].

**Figure 1:**
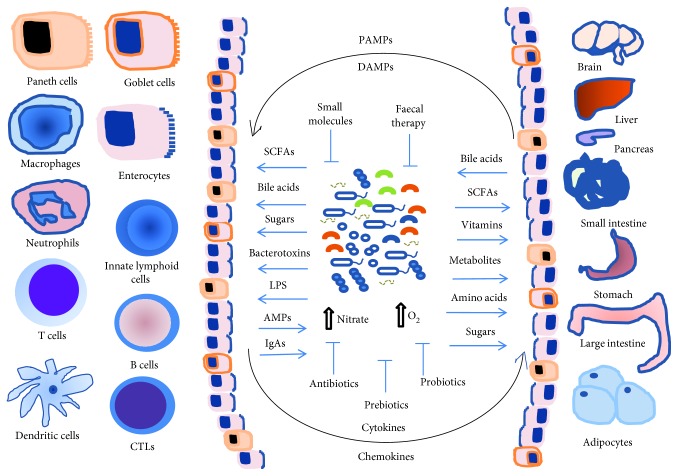
Interplay of gut microbiome-intestinal epithelial cells (enterocytes, goblet cells and paneth cells) and host metabolism and immunity. The commensal, symbiotic, and pathogenic microorganisms provide a great variety of nutrients and metabolites for host metabolism, for energy homeostasis of organs and tissues, and for the innate and adaptive immune cell activation and function. A shift toward dysbiosis results from a decrease in symbiont and/or an increase in pathobiont bacteria in intestinal lumen. Increases in nitrate and oxygen (O_2_) allow the growth of facultative anaerobic bacteria. Increase in the gut permeability and release of PAMPs, such as peptidoglycan and LPS, and DAMPs, such as double-stranded RNA, mtDNA, and ATP. Increase in the production of cytokines, chemokines, nitric oxide (NO), and reactive oxygen species (ROS) by dendritic cells and macrophages causes local and systemic inflammation. Chronic, low-grade systemic inflammation leads to impaired insulin action, insulin resistance, obesity, hypertension, and metabolic syndrome. Probiotics, prebiotics, fecal therapy, and small molecules targeting host genes and specific bacterial species or phylum/class may help to reestablish tissue homeostasis and microbiome healthy. SCFAs: short-chain fatty acids; PAMPs: pathogen-associated molecular patterns; DAMPs: damage-associated molecular patterns; LPS: lipopolysaccharide; AMPs: antimicrobial peptides; IgA: immunoglobulin A.

Figure 1 displays major classes of molecules, metabolites, and nutrients produced by bacterial species, immune cells, tissues, and organs that regulate the dynamic interplay between host cells and gut microbiome, as well as therapeutic strategies for controlling dysbiosis and diseases.

## 4. Immunometabolism and Mitochondrion Reprogramming Pathways

Mitochondria serve as the powerhouse of the cell by producing and releasing critical signals to the environment and synthesizing ATP, the body's energy required for metabolic processes [51]. The bioenergetic pathways of glycolysis, the tricarboxylic acid (TCA) cycle (also known as Krebs cycle and the citric acid cycle), and fatty acid and amino acid metabolism are central metabolic processes for complete oxidation of all nutrients in the mitochondria [51, 52]. Cells use aerobic glycolysis to produce glucose-derived pyruvate that is converted into acetyl coenzyme A (acetyl-CoA). Acetyl-CoA molecules derived from glucose, glutamine, or fatty acid metabolism enter in the TCA cycle and are converted into CO_2_, NADH, and FADH_2_ during oxidative phosphorylation (OXPHOS) OXPHOS occurs through passing electrons along a series of carrier molecules, called the electron transport chain, with the help of electron carriers, such as NAD(P)H and FADH_2_, that serve as substrate to generate adenosine triphosphate (ATP) in the mitochondrial matrix [51, 52]. The electrons are transferred from NADH to O_2_ through three protein complexes: NADH dehydrogenase, cytochrome reductase, and cytochrome oxidase. Electron transport between the complexes occurs through other mobile electron carriers, ubiquinone and cytochrome c. The newly synthesized ATP is transported to the cytosol by adenine nucleotide translocase in exchange for ADP. Acetyl-CoA is the precursor for the synthesis of cholesterol and fatty acids, which are incorporated in the cellular plasma membranes.

**Figure 2:**
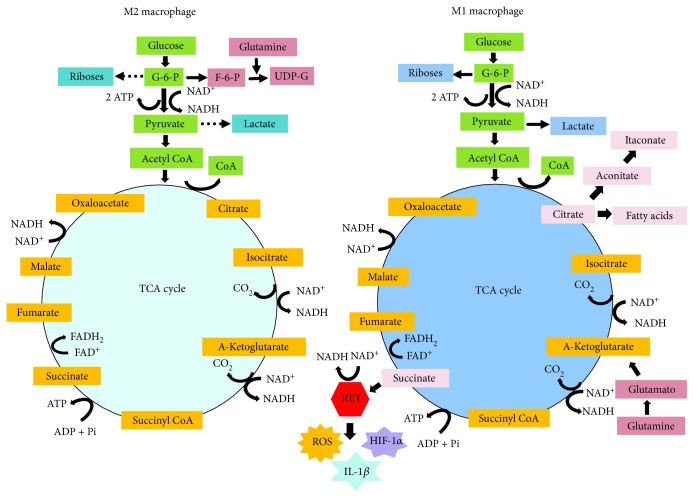
Metabolic pathways supporting macrophage reprogramming in either M2 or M1 phenotype. IL-4-stimulated M2 macrophage utilizes glycolysis, TCA, and mitochondrial oxidative phosphorylation (OXPHOS) to generate NADPH and ATP for energy. G-6-P and glutamine are used for generation of UDP-Glc-Nac required for receptors and protein glycosylation. LPS-stimulated M1 macrophage produces G-6-P that is reduced to pyruvate, in parallel, NAD^+^ is reduced to NADPH, and 2 ATP molecules are produced. In hypoxia, pyruvate is reduced to lactate, restoring NAD^+^ to glycolysis recycle, and thus, OXPHOX is uncoupled to glycolysis. TCA cycle in LPS-stimulated M1 macrophageis deviated after citrate and after succinate. Citrate is used for the syntheses of NO, ROS, and prostaglandins. Citrate is used by the enzyme immune-responsive gene 1 (IRG1) to generate itaconate that acts as an endogenous SDH inhibitor and also as antimicrobial. Succinate is oxidized by SDH and subsequently used for production of ROS from a complex 1 via reverse electron transport (RET). Succinate activates HIF-1*α* which in turn increases the transcription of IL-1*β*. Glutaminolysis provides NADPH for generation of ROS. SDH: succinate dehydrogenase; UDP-Glc-Nac: uridine diphosphate N-acetylglucosamine; NO: nitric oxide; ROS: reactive oxygen species. Arrows indicated the direction of reaction and products.

Immunometabolism comprehends the hub of biochemical activities carry out by immune cells to modulate gene expression profile and switching metabolic pathways and their key enzymes [53]. After a stimulatory signal, various immune cells, in particular, macrophages, DCs, and T cells, exhibit distinct reprogramming metabolic pathways to promote their activation, survival, and lineage generation [53]. This reprogramming in the metabolic pathways is best characterized in macrophages and is illustrated in Figure 2. A seminal study by Newsholme et al. led to the discovery that the consumption rate of glucose, glutamine, and fatty acids and the enzymatic activities of glucose-6-phosphate dehydrogenase, 6-phosphogluconate dehydrogenase, citrate synthase, oxoglutarate dehydrogenase, and glutaminase differ in resting and elicited (inflammatory) macrophages [54]. Their study demonstrated that all glucose utilized by inflammatory macrophages was converted into lactate and very little of it was oxidized [54, 55]. Since then, the shift from oxidative phosphorylation (OXPHOS) toward glycolysis and glutaminolysis has been considered as a metabolic reprogramming pathway of the inflammatory cells. Remarkably, macrophages, T cells, and among other immune cells use glycolysis for rapid production of radical oxygen species (ROS) used for limiting infection. Glycolysis also provides rapid production of ATP and metabolic intermediates for the synthesis of ribose for nucleotides and amino acids for the biosynthesis by RNA and DNA of proteins by macrophages. Glycolysis is a preferential pathway for the activation of dendritic cells, CD4^+^ T helper 1 (Th1), Th2, and Th17 cells, NK cells, and cytotoxic CD8^+^ cells [51, 52]. Interesting, this metabolic adaptation to aerobic glycolysis (in the presence of oxygen) was first described as a hallmark of tumor cells by Warburg et al. in a seminal report published 60 years ago [56].

Unstimulated macrophages displaying M0 phenotype and exhibiting the differentiation surface markers CD68^+^/CD80/^low^/CD206^high^ acquire and display the M1 phenotype (CD68^+^/CD80/^low^/CD206^low^) or M2 phenotype (CD68^+^/CD80^high^/CD206/^low^) after switching their metabolism and function in response to stimuli or polarization signals that initiate a pro- or anti-inflammatory response [57]. M1 cells display Th1-oriented proinflammatory effector properties and promote tissue damage and antimicrobial and antitumor resistance, whereas M2 cells exhibit tissue remodeling and repair functions, promote wound healing, angiogenesis, and resistance to parasites, and favor tumor growth. The reprogramming metabolic pathways in M1/M2 macrophages alter their functions, including cytokine production, phagocytosis, and antigen presentation [58–60]. The classical activation pathway or reprogramming pathway in M1 macrophages promotes the accumulation of citrate and high production of NO, ROS, cytokines, and prostaglandins [60, 61]. M1 macrophages release ROS and NO in phagosomes where they promote the killing of pathogens. Th2 cytokines, such as IL-4 and IL-13, regulate macrophage alternative pathway or M2 reprogramming pathway. The major feature of M2 reprograming pathways is that TCA cycle occurs coupled to oxidative phosphorylation, *β*-oxidation of fatty acids, and mitochondrial biogenesis. In addition, M2 macrophages do not produce NO. It is important to consider that M2 macrophages display various distinct forms (M2a, b, and c) depending on the local tissue environment and exposure to stimuli [57].

The generation of UDP-GlcNAc intermediates promotes the glycosylation of M2-associated receptors, such as the mannose receptor [60]. Thus, both macrophage M1 and M2 phenotypes can be controlled by either oxygen and nutrients or cytokines and damage- and pathogen-associated molecular pattern- (DAMP- and PAMP-) mediated signals [60]. These events are controlled by the signaling and transcriptional pathways induced by canonical regulators of cellular metabolism, such as C-myc transcription factor, coactivator proteins such as PPAR*γ* and PGC-1*β* (peroxisome proliferative-activated receptor *γ*, coactivator 1*β*), and signaling pathways driven by AMPK (5′-adenosine monophosphate-activated protein kinase), mTORC1/2 (mammalian target of rapamycin complexes), and STAT6 (signal transducer and activator of transcription) [51, 52]. A deep overview of macrophage metabolic pathways and phenotypic and functional outcomes is described elsewhere [62].

In 2013, Tannahil et al. reported that expression of IL-1*β* mRNA in M1 macrophages stimulated with LPS leads to activation of the transcription factor hypoxia-induced factor 1*α* (HIF1*α*), in a glycolysis-dependent manner [63]. HIF1*α* interacts with pyruvate kinase isoenzyme M2 (PKM2) and promotes the expression of HIF1*α*-induced genes required for glycolysis [64]. The activation of the Warburg effect (glycolysis) in LPS-stimulated macrophages causes an accumulation of intermediates of the TCA cycle, in particular, succinate, malate, and fumarate due to a flux deviation or a “break point” of the Krebs cycle pathway (see Figure 2). Succinate exerts multiple immunological functions [52, 53, 63]. Oxidation of succinate by the enzyme succinate dehydrogenase (SHD), which converts succinate to fumarate, drives the production of ROS from complex II in the mitochondrion, a process named reverse electron transport (RET). Macrophages respond to activation of HIF-1*α* via ROS and increasing the expression of IL-1*β* [58]. Inhibition of SDH with dimethylmalonate inhibits IL-1*β* expression, while increasing the production of immunosuppressive cytokine IL-10 [52, 63].

Itaconate is one important metabolite formed in the second flux deviation or “break point' of the Krebs cycle pathway during macrophage transition from inactive to proinflammatory state [65]. Itaconate is an unsaturated dicarboxylic acid produced by extra mitochondrial enzyme cys-aconitate decarboxylase encoded by immune-responsive gene 1 (*Irg1*). This enzyme converts cys-aconitate (derived from citrate) to itaconic acid. One of remarkable effect observed after itaconate treatment is the reduction of the expression of cytokines IL-1*β*, IL-6, and iNOS [65]. Studies using murine bone marrow-derived macrophages (BMDMs) and RAW-264.7 cells, stimulated with LPS and cytokines, showed that the absence (knockdown) of *Irg* gene leads to impairment in substrate-level phosphorylation (SLP) of mitochondria [66]. SLP is a metabolic reaction that results in the formation of ATP or GTP by the direct transfer of phosphate group to ADP or GDP from another phosphorylated compound. Administration of itaconate (0.5-2 mM) reverses this reaction [66]. Studies using transgenic mice and immune cells deficient of *Irg* 1 gene demonstrated that itaconate acts as an endogenous succinate dehydrogenase inhibitor and that such inhibition causes the accumulation of succinate [67]. Together, these studies confirmed that itaconate regulates succinate levels, mitochondrial respiration, and inflammatory cytokine production and, therefore, acts as an important regulator of macrophage activation. Furthermore, itaconic acid has antimicrobial activity and kills directly intracellular *Salmonella typhimurium*, *Legionella pneumophila*, and *Mycobacterium tuberculosis*; thus, it protects against infection. The cytotoxic effect is associated with the inhibition of the enzyme isocitrate lyase [68]. Isocitrate lyase regulates a bacterial specific metabolic pathway known as the glyoxylate shunt, in which acetyl-CoA is converted to succinate for the synthesis of carbohydrates. In this pathway, isocitrate is cleaved by the enzyme ICL (encoded by aceA) yielding glyoxylate and succinate, which reenter into TCA cycle following the oxidative decarboxylation steps. Thus, the glyoxylate shunt acts as microbial survival pathway [66–68].

## 5. Gut Dysbiosis Associated with Metabolic Diseases

Studies on the relationship among gut microbes, obesity, insulin resistance, and metabolic syndrome have shown an intricate interplay between host diet, genetics, and microbiome compositional dynamics [49, 69, 70]. A series of experiments have shown that a chronic inflammatory process through translocation of gut bacterial LPS into the bloodstream initiates a silent metabolic endotoxemia and ultimately obesity-related disorders [71–73]. The hallmarks of clinical manifestations of metabolic syndrome include central obesity, high blood pressure, and high levels of blood sugar and serum triglycerides, which are most significant drifts to the development of insulin resistance, type 2 diabetes, hypertension, and fatty liver disease. Individuals robustly colonized by bacteria and archeae of the genera *Faecalibacterium*, *Bifidobacterium*, *Lactobacillus*, *Coprococcus*, and *Methanobrevibacter* have significantly less tendency to develop metabolic disturbances and inflammation and, in turn, type 2 diabetes and ischemic cardiovascular disorders [49, 73]. These species are higher producers of SCFAs and hydrogen peroxides, which are compounds known to inhibit biofilm formation by pathogenic species, including *Staphylococcus aureus* and *E. coli* [73].

Obesity is associated with behavioral and environmental factors, such as excessive consumption of energy-dense foods and sedentary lifestyle [49, 73–75]. Initial clues for the role of microorganisms in energy homeostasis and obesity appeared from the studies using germ-free animals [76]. Several important questions on the complex interaction between host and microbes in pathophysiology of obesity remain answered [49, 74, 75]. Studies in genetic and diet-induced mouse models of obesity confirmed that the ratio of Firmicutes to Bacteroidetes is increased in obese animals, as compared to nonobese control animals [33, 49, 77]. The higher ratio of Firmicutes to Bacteroidetes was also found in clinical studies that evaluated overweight and obese healthy volunteers [33], in high total amount of fecal SCFAs was detected [78]. Mouse and human models have demonstrated inverse relationships between *A. muciniphila* colonization and inflammatory conditions. In fact, *A*. *muciniphila* is present in low levels in people suffering from morbid obesity, diabetes, and cardiometabolic diseases supporting their role as antiobesity strain [79].

The contribution of GI hypothalamic-pituitary-adrenal axis for predisposition to obesity is still incompletely understood [18, 70]. Recently, one study described the relationship of high-fat diet and the levels of acetate produced by intestinal microbiota in a rat model [80]. The authors concluded that chronic acetate turnover activates the parasympathetic nervous system, which coordinates the secretion of glucose-stimulated insulin, ghrelin, and hyperphagia. They conclude that together these factors cooperate in the promotion of obesity [80]. High-fat diet-induced obesity leads also to chronic low-grade hypothalamic inflammation and activation of both microglia and astrocytes [81]. A study using mice model suggests that acetate accumulated in the hypothalamus. This compound plays a central role in prevention of weight gain through an anorectic effect [82]. The neuro-immuno-endocrine pathway is crucial for glucose homeostasis and control of adiposity in obese people recovering from bariatric surgery [80]. After bariatric surgery by Roux-en-Y gastric bypass (RYGB) methods, human and animal models change food preference [83, 84]. Normally, a fatty acid derivative named oleoylethanolamide (OEA) is produced after ingesting a fat meal. OEA can directly activate PPAR-*α* receptors, which are responsible for promoting satiety via vagus nerve and dopamine release in the brain [84]. Dopamine-suppressed obese animals display low levels of OEA in the brain. After RYGB surgery, these animals increase the levels of OEA and dopamine 1 receptor (D1R) expression, which promote a shift in GI-brain axis signaling. This leads to dramatic change in animal feeding behavior which includes the preference for low-fat food [84]. RYGB is also known to profoundly affect the secretion of many gastrointestinal hormones, including ghrelin and GLP-1 [83]. Administration of exogenous GLP-1 or GLP-1 analog promotes weight loss and glucose regulation in T2DM patients. Remarkably, metformin, a drug used to treat T2DM, can increase butyrate-producing bacteria and thereby promote the restoration of the healthy microbiome [85]. Together, these discoveries have opened new perspectives for future therapies to improve health by targeting pivotal host cells, microbes, and microbiome-derived metabolites.

## 6. Targeting Gut Microbiota Dysbiosis and Metabolic Pathways

### 6.1. Probiotics and Prebiotics

The immunologist Elie Metchnikoff was the first to defend that the ingestion of fermented milk (i.e., yogurt) prepared with *Bacillus bulgaricus* increases health and prolong life span. He anticipated the rational use of probiotic made of live microorganisms with a health benefit through altering the gut microbiome [86]. *Lactobacillus plantaram* and *Bifidobacterium* are probiotic bacteria capable of modulating negative effects of high-fat diets and even managing immunological reactions mediated by inflammatory diseases [25, 38, 45]. *Bifidobacterium* and *Lactobacillus* are producers of folate in the gut. *Lactobacillus rhamnosus* in combination with *Lactobacillus gasseri* and *Bifidobacterium lactis* may reduce weight gain, in particular, fat tissue mass adiposity, in humans [87]. Studies on the effects of *A. muciniphila* alive or pasteurized, in high-fat diet-fed mice, revealed that a small 30 kDa Amuc_1100 protein activates TLR2, allowing the development of an intestinal health immune response [79].

Dietary prebiotics are known to provide immune and metabolic benefits to the host [14, 86]. The poorly digestible carbohydrates, such as nonstarch polysaccharides, resistant starch, nondigestible oligosaccharides (NDOs), and polyphenols, are source of various sugars including glucose, galactose, rhamnose, and rutinose. The carbohydrate-hydrolyzing enzymes of colonic microbiota promote the fermentation of prebiotics, and these produce hydrogen, methane, carbon dioxide, and SCFAs. When associated, the probiotic and prebiotic can selectively stimulate growth and activity of health-promoting bacteria [14, 86]. In this way, probiotic and prebiotics have potential to modulate metabolic processes involved in T2DM and obesity-related disorders [14, 86]. Ingestion of inulin or oligofructose improves metabolic disorders associated with obesity, including insulin resistance and metabolic endotoxemia [34, 71, 79]. These effects may be associated with restoration of gut barrier integrity and reduction of LPS release from Gram-negative bacteria [25, 88].

### 6.2. Fecal Microbial Transplant (FMT)

The use of antibiotics permanently modifies gut microbial community [89]. High doses and frequency of antibiotics, particularly against anaerobes, such as vancomycin, can disrupt and destabilize normal gut microbiome. Extended spectrum antibiotic causes the overgrowth of *Clostridium difficile* and chronic recurrent colitis [89]. Fecal bacteriotherapy is a clinical procedure, in which a liquid suspension of stool from a human donor (a family member or a disease-free screened donor) is inoculated into gut's patients to restore gut microbiota in refractory cases of *Clostridium difficile* colorectal infection after antibiotic therapy [90, 91]. These fecal preparations may contain from 3.0% to 10% of viable, dead bacteria and colonic cells and other components that impact on transplant outcomes, by enhancing or inhibiting immune function of innate lymphoid cells (ILCs) [92]. Today, more than 150 clinical trials involving FMT are being conducted, mostly in the USA to treat a large array of metabolic, infectious, and immunological diseases and complications, including kidney- and liver-transplanted patients, especially with antibiotic-resistant bacterial colonization or infection (ClinicalTrials.gov).

Studies have shown that around 90% of the patients that received FMT for *Clostridium difficile* infection were cured, as compared to 31% of those receiving only vancomycin (van Nood et al. [93]). *Lactobacillus*, *Enterococcus*, *Bifidobacterium*, and *Bacteroides* are known to exert an inhibitory activity against *C. difficile* growth. For instance, a variety of viruses, archaea, fungi, parasitic species, and metabolites are transferred together with FMT [92]. Thus, further studies are needed to confirm their ability to potentially promote host intestinal immunity while optimizing microbiome diversity.

A series of animal model studies have shown that the gut microbiome plays a crucial role in weight gain and obesity [49, 50, 70]. Studies on lean and genetically obese (ob/ob) mice and rats have revealed great difference in their intestinal absorption and microbiome composition, which are related to 50% reduction in Bacteroidetes and proportional increases in Firmicutes and Archaea species [74, 75]. Transferring the microbiota from fat mice to germ-free mice hosts induces greater weight gain than in those receiving the microbiota from lean donors [49, 50]. High-fat intake increases intestinal permeability and diffusion of LPS associated with obesity. In contrast, the administration of *Bifidobaterium infantis* in mice reduces the production of proinflammatory cytokines while promoting white adipose tissue gain [68, 71]. Results of FMT treatment of people with diverse metabolic disorders have not been conclusive. Transfer of intestinal microbiota from lean donors mildly increased insulin sensitivity in subjects with metabolic syndrome [94]. Short exposure to antibiotics may improve peripheral insulin sensitivity in a small number of obese subjects [94]. Furthermore, in a study with 75 obese and prediabetic volunteers that underwent an 8-day antibiotic treatment (amoxicillin and vancomycin), the antibiotic-driven dysbiosis did not alter gut permeability, as confirmed by variation in LPS levels and expression of lipid metabolic enzymes [95]. Such heterogeneity may reflect complex interactions between genetic, lifestyle, and environmental factors derived from different model systems.

### 6.3. Small Molecules

Acetate, propionate, and butyrate are the most important SCFAs that eventually are given as oral dietary supplementation. SCFAs acting as signaling molecules produce various physiological effects in humans [96]. They bind to GPR41 and GPR43 receptors (free fatty acid receptors 2 and 3, FFARs 2/3) in intestinal L cells signaling the release of GLP-1 and peptide YY [78]. Notably, injection of GLP-1 peptides stimulates insulin signaling in white adipose tissues, therefore reducing adiposity. SCFAs also increase leptin secretion and adipogenesis while inhibiting lipolysis of adipose tissues. In the liver, propionate acts as a gluconeogenic factor while acetate and butyrate act as lipogenic factors [96]. SCFAs, particularly, propionic and butyric acids, may directly prevent low-grade inflammatory response in obesity by controlling gut microbiota [88]. Butyrate acting on human monocyte, marrow-derived DCs, and macrophages inhibits IL-12 production, decreases costimulatory molecule expression, and blocks NF-*κ*B translocation [37]. Butyrate and structural analog and ketone body *β*-hydroxybutyrate (also known as 3-hydroxybutyrate) supplementation can inhibit the activity of histone deacetylases (HDACs). HDACS promote specific histone modifications to regulate transcription and DNA replication and repair. It also exerts anti-inflammatory activity by suppressing NF-*κ*B and STAT1 activation [22–95]. Succinate is a well-known inhibitor of the histone demethylases (DNMTs) and the eleven translocation (TET) methylcytosine dioxygenases, which oxidize 5-methylcytosines to promote DNA demethylation [22].

Several small molecule inhibitors of metabolic pathways such as 2-deoxy-glucose, dichroacetate, BPTES (bis-2-(5-phenylacetamido-1,3,4-thiadiazol-2-yl)ethyl sulfide), dimethylmalonate (DMM), rotenone, and metformin have identified as promise therapeutics [51, 52, 60]. Metformin is a first-line medication for the treatment of T2D that significantly improves metabolic parameters such as body weight, insulin, glucose, leptin, and C-reactive protein plasma levels [96]. By inhibiting complex I in mitochondrial respiration chain, metformin elevates the plasma levels of lactate. Lactic acidosis is a critical problem in clinical practice. Metformin causes a switch from OXPHOS to aerobic glycolysis [97]. Thus, users of metformin have a higher risk of metformin-associated lactic acidosis. Metformin treatment increases significantly the relative abundance of *A. muciniphila* in the fecal microbiota in obese mice [34]. *A*. *muciniphila* is a producer of acetate and propionate, through mucin degradation, and T2D patients treated with metformin increase their butyrate- and propionate-producing bacteria, which may in turn contribute to the beneficial effect of metformin [85]. It is interesting to ask if metformin effects are also associated with M1/M2 macrophage metabolic reprogramming pathways.

Succinate is a strong proinflammatory mediator [51, 63]. DMM inhibits mitochondrial ROS production through inhibition of succinate dehydrogenase (SDA), which converts succinate to fumarate. This metabolic alteration limits the production IL-1*β*, while increasing the synthesis of immunosuppressive IL-10 [67]. TEPP-46 and DASA-58, two small molecule inhibitors of PKM2 (pyruvate kinase isozyme 2), inhibit LPS-induced HIF-1*α* and IL-1*β*, thereby promoting the reprogramming of M1 macrophages to M2 [63, 98]. Thus, further studies are required to confirm these pharmacological targets and approaches to control metabolic disorders.

## 7. Conclusions and Perspectives

Over the last decade, the sequencing and analyses of a large number of human microbiomes and assembly of their metabolic pathways have expanded our understanding of how bacterial metabolites participate in the microbe-host interactions in health and diseases. The functional variations of gut microbiomes among individuals have indicated the anabolic and catabolic pathways of essential importance in maintaining core community structures for whole body homeostasis. Emerging new biomarkers are promising specific discrimination of phyla and species to confirm evidences for direct participation of microbes in type II diabetes, obesity, metabolic disorders, inflammatory bowel diseases, and even certain cancers.

There is no doubt that dysbiosis, by altering microbiome metabolism and consequently host metabolism, not only affects inflammatory responses and adaptive immunity but also contributes to metabolic disorders. The use of innovative pharmaceutical and nutraceutical products to manage microbial colonization and development of a healthy gut microbial community at early childhood and adult life may prevent the occurrence of common inflammatory and metabolic pathologies. Finally, the discovery and development of drugs that target enzymes of metabolic pathways, and also drive pro- and anti-inflammatory responses of immune cells, will provide the next frontier medicine for metabolic therapies in near future.
